# A series of hydroxychloroquine-associated cardiotoxicity presenting with heart failure

**DOI:** 10.1097/MS9.0000000000002997

**Published:** 2025-02-27

**Authors:** Natalia Nazarenko, Maisha Maliha, Matthew Parker, Pawel Borkowski, Ibolya Csecs, James M. Tauras, Jooyoung Julia Shin, James Pullman, Daniel Sims, Yogita M. Rochlani

**Affiliations:** a Jacobi Medical Center, Bronx, New York, USA; b Dhaka Medical College, Dhaka, Bangladesh; c Yale School of Medicine, New Haven, Connecticut, USA; d Montefiore Medical Center, Bronx, New York, USA

**Keywords:** cardiotoxicity, heart failure, hydroxychloroquine

## Abstract

**Background:**

Hydroxychloroquine (HCQ) is widely used to manage autoimmune conditions such as systemic lupus erythematosus (SLE), rheumatoid arthritis (RA), and Sjogren’s syndrome. However, HCQ can cause cardiotoxicity, a dose-dependent complication linked to the accumulation of metabolites in lysosomes that alters cellular pH. HCQ-induced cardiotoxicity can lead to various cardiac abnormalities, including conduction defects, hypertrophy, and heart failure (HF). Importantly, this toxicity may be reversible with early detection and prompt discontinuation of HCQ.

**Case Summary:**

Three cases of SLE patients with prolonged HCQ use are presented, all of whom exhibited signs of cardiomyopathy and HF. The first case involved a 70-year-old male with a 30-year history of HCQ use, who presented with Mobitz II A-V block and other cardiac abnormalities. The second case was a 45-year-old female with a 26-year history of HCQ therapy who developed sinus tachycardia and biatrial enlargement. The third case involved a 75-year-old woman with 30 years of HCQ use, presenting with shortness of breath and pulmonary hypertension. In all cases, HCQ was discontinued, and supportive HF therapy was initiated, leading to improved ejection fraction and resolution of symptoms within months.

**Discussion:**

HCQ cardiotoxicity, although rare, is an important consideration in patients on long-term therapy, particularly as it is potentially reversible. The diagnosis can be challenging due to the nonspecific nature of cardiac symptoms and overlaps with other conditions. Imaging, particularly cardiac magnetic resonance imaging, plays a crucial role in early detection, while endomyocardial biopsy provides a definitive diagnosis. These cases underscore the need for clinicians to be aware of HCQ cardiotoxicity, as early intervention can improve patient outcomes.

## Introduction

Hydroxychloroquine (HCQ) is a commonly prescribed medication for treating autoimmune diseases, especially systemic lupus erythematosus (SLE), rheumatoid arthritis (RA), and Sjogren’s syndrome (SS). One of the side effects of HCQ is cardiotoxicity, a dose-dependent phenomenon due to the accumulation of metabolites in lysosomes, which subsequently alters cellular pH^[1,2]^. Emerging evidence indicates that HCQ cardiotoxicity can present with several different cardiovascular complications, including conduction abnormalities, biventricular concentric hypertrophy, biatrial enlargement, left ventricle (LV) dysfunction involving impaired LV ejection fraction (EF), and infiltrative cardiomyopathy^[2]^. It is important to note that HCQ-associated cardiotoxicity may be reversible if promptly diagnosed, underscoring the importance of early detection. We depict a series of cases involving patients with SLE and prolonged HCQ use, presenting with symptoms of cardiomyopathy and clinical heart failure (HF), who were subsequently diagnosed with HCQ cardiotoxicity. The work has been reported in line with the PROCESS criteria^[3]^.

**Highlights**

- **Hydroxychloroquine (HCQ)** is commonly used to treat autoimmune conditions like systemic lupus erythematosus (SLE), rheumatoid arthritis, and Sjogren’s syndrome.
- **Cardiotoxicity**, a potential side effect of HCQ, occurs in a dose-dependent manner and can cause a range of heart complications, including conduction abnormalities and left ventricular dysfunction.
- Early detection is critical since **HCQ-associated cardiotoxicity can be reversible** if identified promptly.
- The document outlines cases where patients with SLE developed **heart failure and cardiomyopathy** after prolonged HCQ use and were diagnosed with HCQ cardiotoxicity.

## Case presentation

### Patient #1

**Figure 1.**
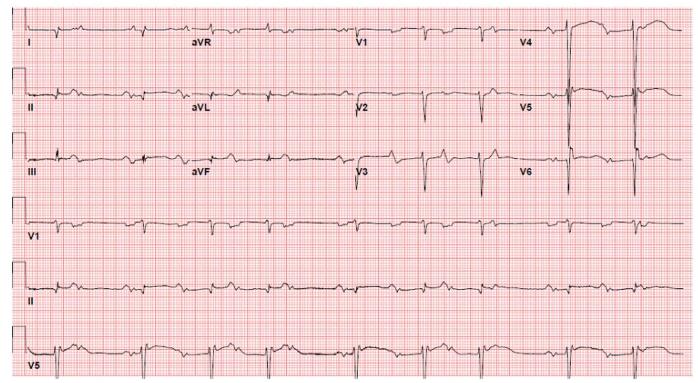
EKG (Patient #1): Sinus rhythm with 2nd-degree A-V block (Mobitz I), premature supraventricular complexes, low QRS voltage in the limb leads, biatrial abnormality, and an old lateral myocardial infarction.

**Figure 2.**
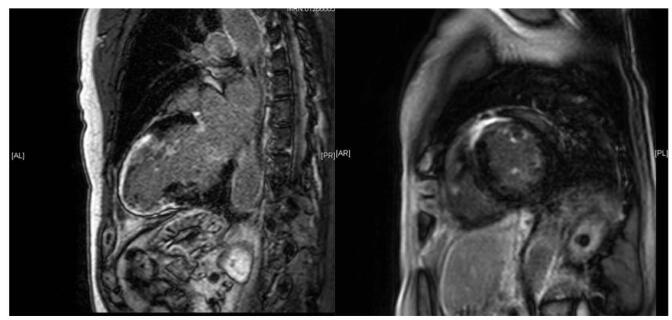
Cardiac MRI (Patient#1): Extensive near transmural LGE in the basal to mid anterior segments extending into all apical segments and subendocardial decreased perfusion at rest, along mid-septal linear mid LGE.

A 70-year-old man presented with complaints of shortness of breath, chest pain, abdominal pain, and bilateral lower extremity edema of 10 days duration. Vital signs were within normal limits, and his physical exam was significant for +1 bilateral pitting edema up to the knees and bibasilar lung rales. He had a past medical history of SLE, diagnosed at age 16, for which he had been prescribed HCQ 200 mg daily for over 30 years. Initial investigations revealed elevated troponin I of 0.70 ng/mL (*n* < 0.03 ng/mL) and B-type natriuretic peptide (BNP) of 2800 pg/mL (*n* < 150 pg/mL). EKG revealed Mobitz II atrioventricular (A-V) block, low QRS voltage in the limb leads, biatrial abnormality, and an old lateral myocardial infarction (Fig. 1). The patient underwent a diagnostic left heart catheterization, which showed non-obstructive coronary artery disease. Transthoracic echocardiography (TTE) and cardiac magnetic resonance imaging (CMR) (Fig. 2) indicated significant abnormalities (Table 1). Pyrophosphate scintigraphy (PYP) scan was unremarkable. Right heart catheterization (RHC) showed normal filling pressures and low cardiac index (CI). Endocardial biopsy (EMBx) revealed typical curvilinear/lamellar bodies with vacuolization (Table 2). Given these findings, HCQ cardiotoxicity was established, HCQ was discontinued, and the patient was started on metoprolol succinate and sacubitril-valsartan. Five months later, the patient did not report any symptoms suggestive of HF, with excellent exercise tolerance and no interim readmissions. Five months after his initial presentation, a repeat TTE showed an EF of 40%.

**Table 1:** Description of TTE and cardiac MRI findings

Investigations	Patient #1	Patient #2	Patient #3
Transthoracic echocardiography	Severe biatrial enlargement, severe concentric left ventricle hypertrophy (LVH) with akinetic apex, left ventricle ejection fraction (LVEF) = 45%, and diastolic dysfunction grade II.	Mild left atrium (LA) dilatation, normal right atrium (RA) size, mild right ventricle (RV) hypokinesis, concentric LVH with severe global LV hypokinesis, LVEF = 15% and diastolic dysfunction grade III.	Severe LA enlargement with severe mitral regurgitation, severe RA enlargement, mild concentric LVH with estimated LVEF = 45–50%, and diastolic dysfunction grade II.
Cardiac MRI	Extensive near transmural late gadolinium enhancement (LGE) involving the basal to mid anterior segments extending into all apical segments with subendocardial decreased perfusion and mid-septal linear and mid-myocardial LGE	Mid-myocardial LGE at the basal inferior/inferolateral walls and severely increased myocardium mass	Diffuse LV hypokinesis with diffuse sub-endocardial LGE with basal predominance

**Table 2:** Hemodynamics and endomyocardial biopsy characteristics of the patients

Parameters	Patient #1	Patient #2	Patient #3
Right atrium (RA)	5	2	3
(*n* = 0–5 mmHg)			
Right ventricle (RV)	20/5	27/5	63/3
(*n* = 10–20 mmHg)			
Pulmonary artery pressure	18/8/11	20/9/14	57/25/36
(*n* < 20 mmHg)			
PCWP (pulmonary capillary wedge pressure)	7	5	25
(*n* = 7–12 mmHg)			
Fick cardiac output (CO)/cardiac index (CI) (*n* = 4–6 LPM/2.5–4 LPM/m^2^	3.2/1.7	3.01/1.81	3.25/2.3
Endocardial biopsy results	Diffuse myocardium fibrosis with a negative Congo red stain on electron microscopy	Unremarkable myocardium with negative Congo red stain and vacuolization and presence of curvilinear/lamellar bodies on electron microscopy.	Multiple myelin bodies Trichrome stain with patchy mild interstitial fibrosis and negative Congo red stain for amyloid on electron microscopy
	Undiagnostic findings on light microscopy.	Undiagnostic findings on light microscopy	Multiple cytoplasmic vacuoles in cardiomyocytes on light microscopy.

### Patient #2

**Figure 3.**
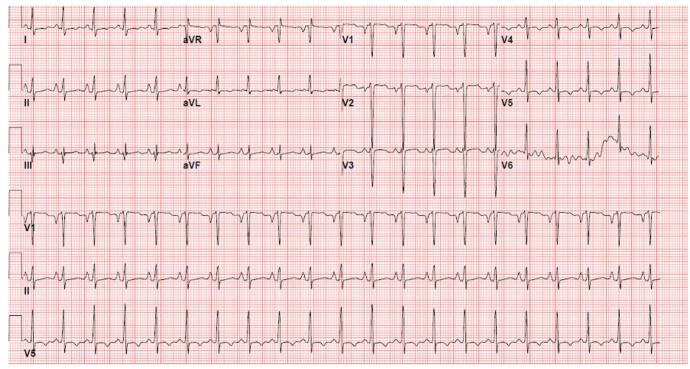
EKG (Patient#2): Sinus tachycardia with biatrial enlargement and negative T-wave inversions in inferior and lateral leads.

**Figure 4.**
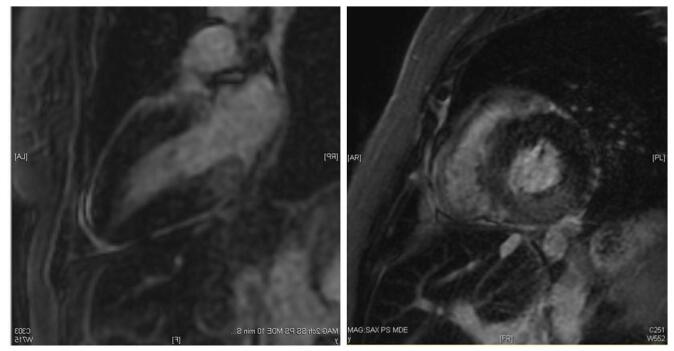
Cardiac MRI (Patient#2): Mid-myocardial LGE at the basal inferior/inferolateral walls.

A 45-year-old female presented to the hospital with complaints of decreased exercise tolerance, exertional dyspnea, and leg swelling of 2 days duration. She appeared restless, with coarse bilateral breathing sounds, orthopnea, +2 pitting edema of the lower extremities, and sinus tachycardia with a heart rate of 145 beats per minute. The patient had a past medical history of SLE, RA, and SS since age 19, for which the patient had been taking HCQ 200 mg daily for over 26 years. Initial troponin I was elevated at 0.64 ng/mL and BNP >5000 pg/mL. Initial EKG revealed sinus tachycardia with biatrial enlargement and T-wave inversions in anterolateral leads (Fig. 3). Diagnostic LHC revealed mild non-obstructive CAD. TTE and CMR (Fig. 4) revealed significant changes (Table 1). After diuresis, RHC showed normal filling pressures and low CI. EMBx showed vacuolization suggestive of HCQ cardiotoxicity, confirmed by electron microscopy, which showed curvilinear/lamellar bodies (Table 2). Guideline-directed medical therapy (GDMT) of sacubitril-valsartan and metoprolol succinate were commenced, and HCQ was suspended. The patient was followed up 3 months later, at which time she displayed an improved EF at 45% and no additional admissions associated with HF exacerbation.

### Patient #3

**Figure 5.**
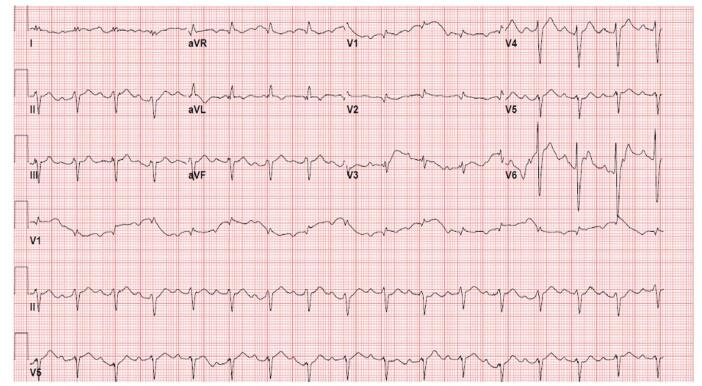
EKG (Patient#3): Sinus rhythm with 1st-degree AV block. Left atrial enlargement. Incomplete right bundle branch block. Left anterior fascicular block.

**Figure 6.**
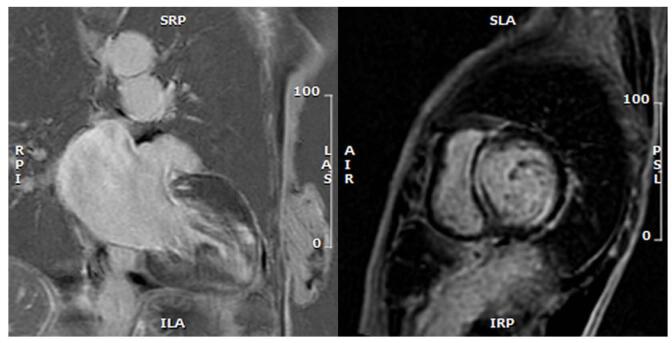
Cardiac MRI (Patient#3): Diffuse sub-endocardial LGE with basal predominance.

A 75-year-old woman presented to the hospital with shortness of breath and palpitations of 3 days duration. Vital signs were normal, and the physical exam was remarkable for bilaterally decreased breathing sounds. The patient was known to have SLE and autoimmune hepatitis and was on HCQ 200 mg (400 mg on alternate days) twice daily for more than 30 years. BNP was 850 pg/mL with high sensitivity troponin of 371 ng/L (*n* = <7 ng/L). Further workup included the following: EKG (Fig. 5); LHC – showing minimal non-obstructive CAD; TTE (Table 1) and CMR (Fig. 6).

**Figure 7.**
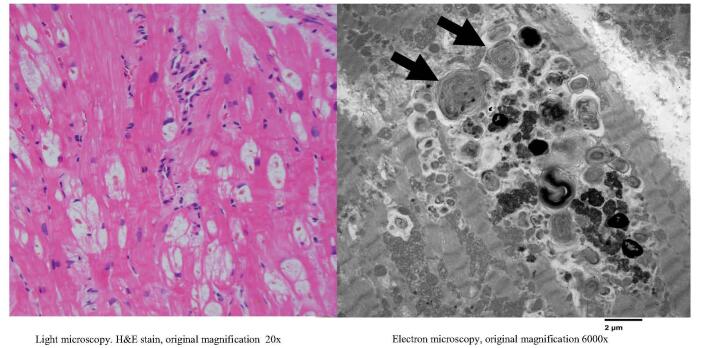
Left side – light microscopy of patient #3 showing multiple cytoplasmic vacuoles (white areas) in cardiomyocytes. Right side – electron microscopy of patient #3 showing multiple myelin bodies displacing myofibrils in myocardial sarcoplasm. Arrows identify the two largest, which show the characteristic concentric multilayering.

PYP scan was negative for amyloidosis of transthyretin type amyloidosis. RHC showed elevated pulmonary capillary wedge pressure with pulmonary venous hypertension and mildly depressed CI. EMBx (Table 2) and light microscopy (Fig. 7) revealed extensive cardiomyocyte vacuolization with myelin bodies. HCQ was discontinued, and the patient started on metoprolol succinate. The patient was followed as an outpatient where a significant improvement in exercise tolerance and the absence of leg edema was reported. No readmissions were noted 6 months after initial admission. A repeat TTE demonstrated an EF of 55%.

## Discussion

HCQ presents as a rare and potentially reversible etiology of cardiomyopathy and HF, making quick diagnosis both difficult and of critical importance. HCQ is frequently used in treating rheumatologic diseases, given its track record of safety, efficacy, and tolerability^[1]^. HCQ has a long half-life of 6 months due to its hydrophobic structure. Moreover, it binds to phospholipids within lysosomes, causing dysregulation of degradation processes and accumulation of toxic metabolites in the form of intracellular vacuoles as seen on light microscopy and curvilinear bodies as seen on electron microscopy imaging^[2]^. A recent study showed that HCQ initiates mitochondrial apoptosis singling, causing cardiomyocyte dysfunction^[4]^.

The incidence of HCQ cardiotoxicity is not very well known as it has only been described in case reports and series. It is reported to have a female predominance^[5,6]^ and is typically seen in patients between 30 and 80 years of age^[2,6]^. The median duration of treatment with HCQ-associated cardiotoxicity has been reported to be 7 years (3 days to 35 years)^[5]^. In our series of three patients, the average duration of HCQ treatment was 30 years, resulting in an estimated cumulative HCQ dose of 2000–2500 g.

A systematic review of over 150 cases of antimalarial-induced cardiomyopathy identified decompensated HF, syncope^[6]^, and conduction abnormalities^[5]^ as the most common clinical presentations. Conduction abnormalities occur due to the infiltrative nature of this cardiomyopathy^[2]^ and are usually progressive. They generally follow a pattern beginning with a right bundle branch block, followed by a left anterior fascicular block, and then a complete heart block^[2]^, with about 50% of patients requiring a permanent pacemaker^[6]^. In our cases, the patients displayed various degrees of conduction abnormalities. These EKG findings may be the first indication of HCQ cardiotoxicity in the appropriate clinical settings. D’Andrea *et al* performed the analysis of 54 462 patients with RA on HCQ vs. methotrexate therapy and found that the HCQ group had a 29% increased risk of HF hospitalizations^[7]^. Imaging features of HCQ toxicity on TTE include LV systolic dysfunction, restrictive diastolic function, LV hypertrophy, biatrial enlargement, and hypertrophy of the interventricular septum. At the same time, CMR findings suggest patchy late gadolinium enhancement (LGE) in a non-coronary artery distribution^[6]^. In our cases, CMR was a pivotal investigative tool, guiding the decision to proceed with myocardial biopsy. In all three patients, the presence of LGE was identified either in the sub-endocardial or mid-myocardial zones, in the absence of obstructive CAD, raising concerns about the potential for toxic versus infiltrative heart disease. The resolution of CMR abnormalities has been reported with the cessation of HCQ therapy^[5]^, suggesting that CMR can be a potentially helpful tool for follow-up^[6]^. EMBx is the gold standard for establishing the diagnosis, and characteristic histopathology features include intracellular vacuolization on light microscopy and curvilinear bodies on electron microscopy^[2]^.

In our work, we demonstrated how stopping HCQ in a timely manner can improve or reverse associated underlying cardiomyopathy, reduce additional HF admissions, and relieve resulting symptoms, supported by similar descriptions in the literature. Given the widespread usage of HCQ in the context of chronic autoimmune diseases, it is essential to be aware of potential associated cardiotoxicity and maintain a proactive approach in diagnosis and treatment to achieve better clinical outcomes.

## Conclusion

HCQ-associated cardiotoxicity is a rare condition that can potentially be reversed.

It should be considered in patients with recurrent HF exacerbations without clear causes after prolonged HCQ use, particularly when concentric LVH and a negative PYP scan are present, with endomyocardial biopsy serving as the definitive diagnostic tool.

## Data Availability

Not applicable.
